# Suppressor of Cytokine Signaling 2 Regulates Retinal Pigment Epithelium Metabolism by Enhancing Autophagy

**DOI:** 10.3389/fnins.2021.738022

**Published:** 2021-11-08

**Authors:** Xi-Yuan Liu, Rui Lu, Jing Chen, Jie Wang, Hong-Mei Qian, Gang Chen, Rong-Han Wu, Zai-Long Chi

**Affiliations:** State Key Laboratory of Ophthalmology, Optometry and Visual Science, Eye Hospital and School of Ophthalmology and Optometry, Wenzhou Medical University, Wenzhou, China

**Keywords:** suppressor of cytokine signaling 2, retinal pigment epithelium, autophagy, ubiquitin, glycogen synthase kinase (GSK)-3β, mammalian target of rapamycin (mTOR)

## Abstract

Retinal pigment epithelium (RPE) serves critical functions in maintaining retinal homeostasis. An important function of RPE is to degrade the photoreceptor outer segment fragments daily to maintain photoreceptor function and longevity throughout life. An impairment of RPE functions such as metabolic regulation leads to the development of age-related macular degeneration (AMD) and inherited retinal degenerative diseases. As substrate recognition subunit of a ubiquitin ligase complex, suppressor of cytokine signaling 2 (SOCS2) specifically binds to the substrates for ubiquitination and negatively regulates growth hormone signaling. Herein, we explore the role of SOCS2 in the metabolic regulation of autophagy in the RPE cells. *SOCS2* knockout mice exhibited the irregular morphological deposits between the RPE and Bruch’s membrane. Both *in vivo* and *in vitro* experiments showed that RPE cells lacking *SOCS2* displayed impaired autophagy, which could be recovered by re-expressing SOCS2. SOCS2 recognizes the ubiquitylated proteins and participates in the formation of autolysosome by binding with autophagy receptors and lysosome-associated membrane protein2 (LAMP-2), thereby regulating the phosphorylation of glycogen synthase kinase 3β (GSK3β) and mammalian target of rapamycin (mTOR) during the autophagy process. Our results imply that SOCS2 participates in ubiquitin-autophagy-lysosomal pathway and enhances autophagy by regulating GSK3β and mTOR. This study provides a potential therapeutic target for AMD.

## Introduction

Retinal pigment epithelium (RPE) serves critical functions in maintaining retinal homeostasis. Impairment of RPE functions such as metabolic regulation leads to the development of age-related macular degeneration (AMD). An important function of RPE is to digest the photoreceptor outer segment (POS) fragments daily to maintain photoreceptor function and longevity throughout life. RPE undergoes various changes during aging, one of which is weakened autophagy (Giaime et al., 2017; Madeo et al., 2019). The disorder of RPE clearing POS fragments contributes to the accumulation of the intracellular residual bodies, eventually leading to the formation of drusen and development of AMD. AMD, a chronic disease of the macula, is the leading cause of irreversible blindness in the elderly individuals with a steep increase in the number of patients over age 65 years (Mitchell et al., 2018). Atrophic or dry AMD and neovascular or wet AMD are the two forms of AMD. To date, the treatments are limited to wet AMD and no effective treatment exists for dry AMD because the mechanism underlying AMD is still not fully understood. Thus, the discovery of a mechanism is critically needed to prevent or delay AMD progression. Reduced autophagy of RPE with age is one of the risk factors and accelerates the formation of drusen, which is composed of proteins, lipids, and minerals (Wang et al., 2010; Crabb, 2014). Proteomic analysis of drusen identified 129 proteins, most of which were insoluble and aggregate (Crabb et al., 2002; Hollyfield et al., 2003; Ohno-Matsui, 2011). Therefore, enhancing the degradation of metabolic proteins in RPE is one way to maintain retinal homeostasis.

Autophagy is essential for an organism as it improves protein and organellar quality control and helps to defer the age-related phenotypes (Hansen et al., 2018; Nakamura et al., 2019). Mammalian target of rapamycin (mTOR) is inactivated during autophagy initiation and plays a crucial role in regulating autophagy by directly interacting with or inhibiting autophagy-related genes (Kim and Guan, 2015; Saxton and Sabatini, 2017). Autophagy involves not only bulk autophagy but also selective autophagy. Ubiquitin acts as a specificity factor for selective autophagy and the ubiquitin-proteasome system (UPS). UPS is inclined to the rapid degradation of short-lived proteins in the young cells and autophagy selectively removes the long-lived protein aggregates and damaged or excess organelles during aging (Bence et al., 2001; Ravikumar et al., 2002; Gamerdinger et al., 2009). Various metabolic components ranging from protein aggregates, peroxisomes, ribosomes, and mitochondria can be specifically engulfed by autophagosomes through ubiquitination and ubiquitin-binding receptors such as sequestosome 1 (SQSTM1/p62, hereafter referred to as p62), neighbor of BRCA1 gene 1 (NBR1), nuclear dot protein 52 (NDP52), optineurin (OPTN), and toll-interaction protein (TOLLIP) (Kirkin et al., 2009; Thurston et al., 2009; Lu et al., 2014; Yamano and Youle, 2020). p62 serves as a scaffold for the formation of protein aggregates and acts as an autophagy receptor by linking the ubiquitin-tagged protein aggregates to autophagosomes (Dantuma and Bott, 2014; Parzych and Klionsky, 2014; Rogov et al., 2014; Zaffagnini et al., 2018). The ubiquitylated substrates are specifically degraded by a p62-dependent mechanism (Pankiv et al., 2007; Kim et al., 2008). Thus, it is likely that p62 acts as an adaptor between ubiquitylated proteins and autophagy.

Suppressor of cytokine signaling (SOCS) family proteins [SOCS 1–7 and cytokine inducible SH2-containing protein (CIS)] are essential regulators of cellular responses to cytokines *via* the regulation of Janus kinases/signal transducers and activator of transcription proteins signaling. SOCS2 expression is rapidly induced upon cytokine stimulation such as growth hormone (GH) and insulin-like growth factor (Turnley, 2005; Sarajlic et al., 2019). SOCS2 possesses an Src homology 2 (SH2) domain and a SOCS box, which is responsible for E3 ligase activity by assembly with the adaptors Elongin BC and Cullin 5 (Cul5) (Bulatov et al., 2015; Song et al., 2016; Chhabra et al., 2018). The Elongin BC-Cul5-SOCS box complex is stimulated by circulating GH and regulates GH receptors (GHR) through a negative feedback loop (Vesterlund et al., 2011). Glycogen synthase kinase 3β (GSK3β) is inhibited by serine phosphorylation in response to insulin or growth factors (Ge et al., 2013; Wei et al., 2021). GSK3β mediates the activation of mTOR by Wnt signaling and the inhibition of GSK3β increases the activation of mTOR (Inoki et al., 2006). GSK3β induces autophagy by phosphorylating unc-51 like autophagy activating kinase 1 (ULK1) in the adult hippocampal neural stem cells (Ryu et al., 2021). Inhibition of long non-coding RNA (lncRNA) X-inactive specific transcript (XIST) improves myocardial ischemia/reperfusion (I/R) injury by targeting miR-133a through inhibition of autophagy and regulation of SOCS2 (Li et al., 2019). SOCS2 is shown to be upregulated in Huntington’s disease and involved in regulating autophagy by functioning as an E3 ligase (Cho et al., 2021). Another study indicates that the interaction between microtubule associated protein 1 light chain 3 (LC3) and SOCS2 was detected in astrocytes either in normal or in starvation conditions (Wang et al., 2014). The genome-wide association studies discover that SOCS2 is associated with visual loss belonging to vascular endothelial growth factor (VEGF)-related pathways in the patients with exudative AMD (Akiyama et al., 2018). Integrated bioinformatics analysis indicates that hypermethylated and low-expressed SOCS2 is related to AMD (Shen et al., 2020). In RPE, however, the role of SOCS2 in autophagy is largely unknown.

We report the function of SOCS2 on autophagy. During autophagy, SOCS2 colocalizes with ubiquitylated proteins, p62, lipidated LC3B, and lysosome-associated membrane protein2 (LAMP2) and regulates the phosphorylation of GSK3β and mTOR. Our results imply that SOCS2 may participate directly in the ubiquitin-autophagy-lysosomal pathway and enhance autophagy by regulating GSK3β and mTOR.

## Materials and Methods

### Antibodies, Plasmids, and Chemicals

Anti-SOCS2 (#2779), β-actin (8H10D10) (#3700), phosphorylated mTOR (p-mTOR) (Ser2448) (#5536), mTOR (7C10), rabbit monoclonal antibody (mAb) (#2983), goat antirabbit immunoglobulin G (IgG) [horseradish peroxidase (HRP) linked] (#7074), and horse antimouse IgG (HRP linked) (#7076) were purchased from the Cell Signaling Technology (Danvers, Massachusetts, United States); anti-SQSTM1/p62 antibody (ab155686), anti-ubiquitin antibody (ab7254), anti-LAMP2 antibody-lysosome marker (ab25631), recombinant anti-GSK3β antibody (Y174) (ab32391), anti-GSK3β (phospho Y216 and Y279) (ab75745), and recombinant anti-LC3B antibody (ab192890) were purchased from the Abcam (Discovery Drive Cambridge Biomedical Campus, Cambridge, United Kingdom). Antiadvanced glycation end product (AGE) carboxymethyl-lysine (CML) (MABN1837) was purchased from the Millipore (Billerica, MA, United States). Plasmid cytomegalovirus 3 (pCMV3)-Human-SOCS2-orange fluorescent protein (OFP) expression plasmid (HG11285-ACR), control vector OFP expression plasmids (CV025), pCMV3-Human-SOCS2-green fluorescent protein (GFP) (HG11285-ACG), and control vectors GFP expression plasmids (CV026) were purchased from the Sino Biological Incorporation (Wayne, PA, United States). The autophagy inhibitor chloroquine (CQ) diphosphate (c6628) was purchased from Sigma-Aldrich (St Louis, Mosby, United States).

### Animals

All the animal studies were conducted according to the protocols approved by the Institutional Animal Care and Use Committee of Wenzhou Medical University and followed the Association for Research in Vision and Ophthalmology (ARVO) Statement for the Use of Animals in Vision Research and were in accordance with the approved institutional guidelines and regulations. C57BL/6 mice were purchased from the Vital River Laboratories (Beijing, China). SOCS2^–/–^ mice were generated from C57BL/6 mice by using clustered regularly interspaced short palindromic repeats (CRISPR)/CRISPR-associated 9 (Cas9)-mediated genome engineering technology to delete the fragment of exon 3 by using gRNA1 (TTG GCA GTC GTT TTT CTA GT CGG) and gRNA2 (ATT CAG CTA AAA CTA CCT AA GGG) by the Cyagen Biosciences (Guangzhou, China). The mice were kept in the standard cages and fed *ad libitum*.

### Cell Culture and Transfection

The adult retinal pigment epithelium-19 (ARPE-19) cells (CRL-2302) were purchased from the American Type Culture Collection (ATCC) (Manassas, VA, United States) and maintained in Dulbecco’s Modified Essential Medium (DMEM)/F12 (011721ACS) (Boehringer Ingelheim, Shanghai, China) with 10% fetal bovine serum (FBS) (10099, Thermo Fisher Scientific, Wilmington, DE, United States), 100 units/ml penicillin and streptomycin (10378016, Thermo Fisher Scientific, Wilmington, DE, United States) at 37°C with 5% CO_2_. For transfection, ARPE-19 cells reached 80% confluence. SOCS2 plasmids and control vectors were transfected with the Lipofectamine 3000 Transfection Kit (L3000015, Thermo Fisher Scientific, Wilmington, DE, United States) according to the instructions of the manufacturer.

### Isolation and Primary Culture of Murine Retinal Pigment Epithelium

Primary RPE cells were isolated from wild-type (WT) C57BL/6 or SOCS2^–/–^ mice at age of 6 weeks following the previously described method (Fernandez-Godino et al., 2016; Chinchilla et al., 2021). The single-layer RPE sheets were isolated and incubated in DMEM/F12 medium with 15% FBS at 37°C with 5% CO_2_.

### Real-Time Quantitative PCR

Total RNA was extracted by using TRIzol reagent (10296010, Invitrogen, Carlsbad, CA, United States) following the RNA preparation method and was quantified by using the Multiskan Go (Thermo Fisher Scientific, Wilmington, DE, United States) (Brown et al., 2018). Reverse transcription PCR was performed by using the GoScript Reverse Transcription System (A5001, Promega, Madison, WI, United States) following the instructions of the manufacturer. Quantitative real-time PCR was used to detect the messenger RNA (mRNA) expression of SOCS2 by using the iTaq^TM^ Universal SYBR^®^ Green Supermix (172-5121, Bio-Rad, Philadelphia, PA, United States). The primers were listed as follows: mSOCS2-F (5′-3′ CTC AGT CAA ACA GGA TGG TAC T); mSOCS2-R (5′-3′ TAC TCA ATC CGC AGG TTA GTC); mGAPDH-F (5′-3′ GAG CCA AAA GGG TCA TCA TCT C); and mGAPDH-R (5′-3′ GTG AGC TTC CCG TTC AGC TCT); hSOCS2-F (5′-3′) GAGCTCGGTCAGACAGGATG; hSOCS2-R (5′-3′) AGTTGGTCCAGCTGATGTTTT; hGAPDH-F: ATC GTG GAA GGA CTC ATG ACC ACA; and hGAPDH-R: AGA GGC AGG GAT GAT GTT CTG GA. Real-time PCR was performed on a Q5 Real-Time PCR System (Applied Biosystems, Carlsbad, CA, United States). The expression of SOCS2 was calculated by comparing the threshold cycle numbers.

### Duolink *in situ* Fluorescence

Cells on the glass slides were fixed with 4% paraformaldehyde (PFA) for 20 min and the experiment was performed by following the instructions of the manufacturer of Duolink *in situ* fluorescence (DUO92101-1KT, Sigma-Aldrich, St Louis, Mosby, United States). Cells of the experimental group were incubated with SOCS2 and ubiquitin or LAMP2 antibodies and cells of the negative group were incubated without antibodies. Mount slides with a minimal volume of Duolink *in situ* mounting medium with 4′,6-diamidino-2-phenylindole (DAPI) for 15 min before analyzing in the microscope.

### Immunoblot

Retinal pigment epithelium cells were serum starved and treated with or without 30 μM CQ for 24 h. Immunoblots were performed as instructed in ECL immunoblot kits (1705061, Bio-Rad, Philadelphia, PA, United States). Protein bands were exposed and captured by using an imaging system (ChemiDoc XRS+, Bio-Rad, Philadelphia, PA, United States) and quantified by the ImageJ software (United States National Institutes of Health, Bethesda, MD, United States).

### Immunofluorescent Staining

Retinal pigment epithelium cells were serum starved and treated with or without 30 μM CQ for 24 h. Immunofluorescence staining of LC3B, p62, ubiquitin, and LAMP2 was performed and images were captured under a confocal microscope (ZEISS, LSM710, Oberkochen, Germany) as described (Li et al., 2021).

### Autophagic Flux of Retinal Pigment Epithelium *in vivo*

Adeno-associated virus (AAV) type 2 vectors with a tandem monomeric red fluorescent protein (mRFP)-GFP-LC3 construct (AAV2-mRFP-GFP-LC3, HB-AP2100001) were generated by the Hanbio Corporation Ltd. (Shanghai, China). AAV2-mRFP-GFP-LC3 was injected subretinally through the posterior part of the sclera as previously described (Ross et al., 2021). An eye of one adult mouse received 2 μl of the virus at a concentration of 1 × 10^12^ vg/ml. 2 weeks later, RPE/choroidal flat mounts were performed under a microscope. The fluorescence of the cells was visualized under a microscope (DMi8, LEICA, Wetzlar, Germany).

### Histology

Eyes were enucleated from euthanized 1-year old mice, fixed in 4% paraformaldehyde solution, and embedded in paraffin. Tissue sections (5 μm thick) were stained with H&E before imaging. Images of the retina/RPE/choroid were collected.

### Electron Microscopy

Eyes from 1-year old WT and SOCS2^–/–^ mice were fixed in phosphate-buffered saline (PBS)-buffered glutaraldehyde (2.5%, pH 7.4). The cornea, iris, and lens were discarded. The retina/RPE/choroid was fixed one more time in PBS-buffered glutaraldehyde and PBS-buffered osmium tetroxide (0.5%). The retina/RPE/choroid was embedded in epoxy resin. Thin sections (90 nm) were collected on 200 μm mesh copper grids, dried for 24 h, and double-stained with uranyl acetate and lead citrate. Sections were viewed and imaged at 80 kV with a transmission electron microscope (Hitachi-7500, Hitachi Limited, Tokyo, Japan).

### Statistical Analysis

The two-tailed unpaired *t*-test and the one-way ANOVA (GraphPad Prism, CA, United States) were used to calculate the *p*-value of the results. The data are expressed as the mean ± SEM. At least six mice including males and females were used in each group.

## Results

### Suppressor of Cytokine Signaling 2^–/–^ Mice Exhibit Abnormal Retinal Pigment Epithelium-Bruch’s Membrane-Choriocapillaris Complex

**FIGURE 1:**
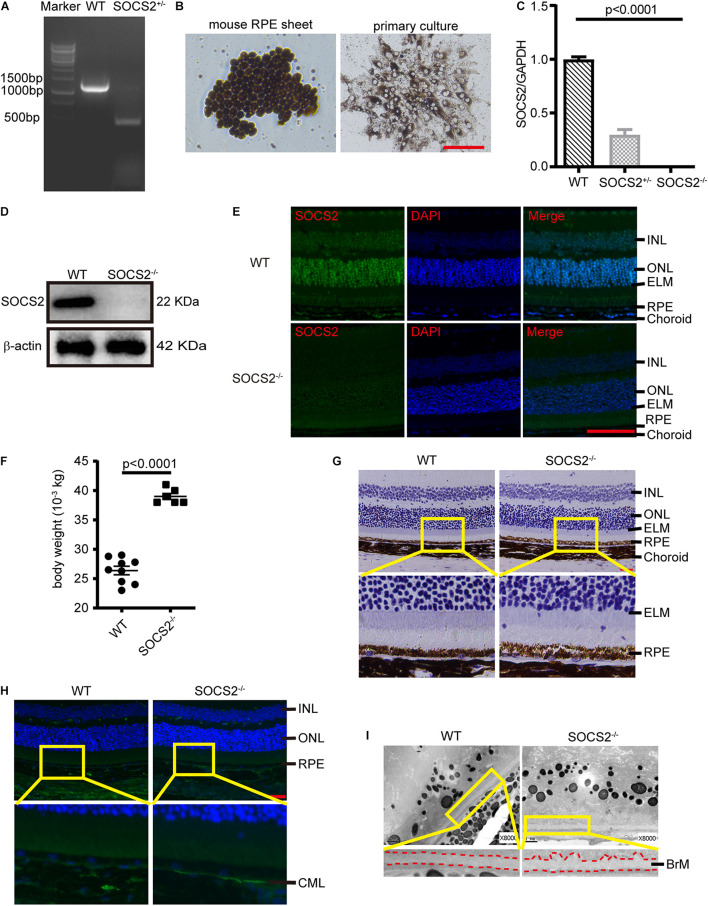
Abnormal RPE-Bruch’s membrane (BrM)-choriocapillaris complex of SOCS2^–/–^ mice. **(A)** PCR screening of SOCS2^+/–^ mice F1 founder to detect SOCS2 sequence from genomic DNA. **(B)** Primary culture of RPE cells isolated from WT mice. Scale bar, 200 μm. **(C)** Real-time quantitative PCR detected the mRNA expression of SOCS2 in the primary RPE of WT, heterozygote, and homozygote. Statistical significance was determined by using the one-way ANOVA. **(D)** Western blot detected the protein expression of SOCS2 in the RPE of WT and SOCS2^–/–^ mice. **(E)** SOCS2 expression and location in the retina of WT and SOCS2^–/–^ mice. Scale bar, 50 μm. **(F)** The body weights of the SOCS2^–/–^ (*n* = 6, 8 weeks old, including males and females) and WT littermates (*n* = 9, 10 weeks old, including males and females). **(G)** The structures of the retinal-RPE-choroid were detected by histology in WT and SOCS2^–/–^ mice (*n* = 6, 1-year old, including males and females). Scale bar, 50 μm. **(H)** CML was detected by immunofluorescence on the sections of retinal-RPE-choroid from WT and SOCS2^–/–^ mice (*n* = 6, 1-year old, including males and females). Scale bar, 50 μm. **(I)** BrM and deposits were detected by transmission electron micrographs on the sections of retinal-RPE-choroid from WT and SOCS2^–/–^ mice (*n* = 6, 1-year old, including males and females). Scale bar, 1 μm. Abbreviations: SOCS2, suppressor of cytokine signaling 2; mRNA, messenger RNA; INL, inner nuclear layer; ONL, outer nuclear layer; ELM, external limiting membrane; RPE, retinal pigment epithelium; WT, wild type.

Mice lacking SOCS2 exhibit gigantism, thickening dermis, and shortening lifespan (Casellas and Medrano, 2008; Gossing et al., 2021). To understand the biological function of SOCS2, SOCS2^–/–^ mice were generated by deleting a 756-bp fragment in exon 3 through CRISPR/Cas9 technology (Figure 1A). The monolayer RPE sheets composed of pigmented and hexagonal RPE were isolated from mice as previously described (Fernandez-Godino et al., 2016) and RPE cells were cultured to obtain a large number of cells (Figure 1B). The expression of SOCS2 mRNA was confirmed in primary cultured RPE from WT, heterozygous, and homozygous mice (Figure 1C), while the protein expression of SOCS2 vanished in the RPE of SOCS2^–/–^ mice (Figure 1D). Immunofluorescence of SOCS2 also confirmed that no SOCS2 expression takes place in the retina of SOCS2^–/–^ mice (Figure 1E). SOCS2^–/–^ mice weighted significantly (1.5-fold) more than their WT littermates at 10-week age (Figure 1F). For the study of fundus phenotypes accumulating with age, mice were selected from 1 year of age. More generally, the abnormal structures of retina were observed in SOCS2^–/–^ mice such as the disorder of the nuclear arrangement of the outer nuclear layer (ONL), the unsmooth external limiting membrane, and the loose structure of the RPE (Figure 1G). The accumulation of CML adducts in Bruch’s membrane was observed in SOCS2^–/–^ mice (Figure 1H). CML is an AGE that results from oxidative stress and chemical glycation and accumulates with age in Bruch’s membrane and drusen in AMD (Hariharan et al., 2011; Kim et al., 2013). SOCS2^–/–^ mice showed thickened Bruch’s membrane and irregular morphological deposits between the RPE and Bruch’s membrane (Figure 1I).

### Suppressor of Cytokine Signaling 2^–/–^ Retinal Pigment Epithelium Exhibits Impaired Autophagy

**FIGURE 2:**
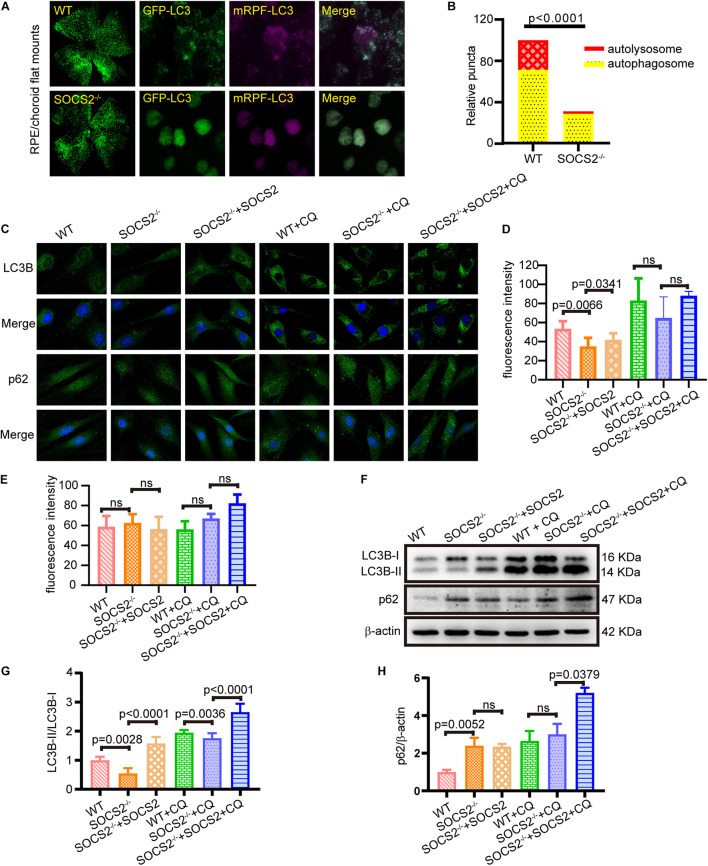
RPE lacking the *SOCS2* gene exhibited impaired autophagy. **(A)** *In vivo*, autophagic flux was detected by injecting 2 μl of 1 × 10^12^ vg/ml adeno-associated virus 2 (AAV2)-mRFP-GFP-LC3 in the eyeball of adult WT and SOCS2^–/–^ mice subretinally. About 2-weeks later, mice were starved for 24 h before euthanasia, and RPE/choroid flat mounts were isolated from mice (*n* = 8, 10-week old, including males and females). **(B)** The numbers of autophagosomes and autolysosomes were evaluated by counting yellow and RFP dots. Puncta dots per horizon are shown as the mean ± SEM. **(C)** LC3B puncta dots (green) and p62 puncta dots (green) were stained by immunofluorescence in mouse primary RPE cells that were starved and treated with or without 30 μM CQ for 24 h. One group of SOCS2^–/–^ RPE re-expressed SOCS2 by transient transfection. **(D,E)** Fluorescence intensity of LC3B and p62 were quantified by the ImageJ software. **(F)** Immunoblotting for LC3B and p62 was performed in mouse primary RPE with different treatments. **(G)** The ratio of LC3B-II/LC3B-I was quantified by ImageJ software and normalized to β-actin. **(H)** p62 was quantified by ImageJ software and normalized to β-actin. Values for the control were set to 1. Statistical significance was determined by using the two-tailed unpaired *t*-test.

Impaired autophagy leads to the progressive accumulations of proteins and other metabolites in the RPE, which, in turn, results in RPE toxicity and pathological conditions. To monitor the *in vivo* autophagic flux of RPE, AAV2-mRFP-GFP-LC3 was injected subretinally into adult WT and SOCS2^–/–^ mice. The differentiation between autophagosomes and autolysosomes was captured by the tandem fluorescent mRFP-GFP-LC3 from RPE/choroid flat mounts (Ranieri et al., 2018). The number of autophagosomes could be evaluated by counting the yellow dots overlapped by GFP and mRFP. Green fluorescence is quenched in the acidic pH of the lysosome. The mRFP dots indicated autolysosome formation. More autophagosomes (yellow dots) and autolysosomes (RFP dots) were present in the RPE of WT mice compared to the RPE of SOCS2^–/–^ mice (Figures 2A,B), suggesting that autophagy was impaired in the RPE of SOCS2^–/–^ mice.

To further evaluate the autophagic activity of RPE from SOCS2^–/–^ mice, *in vitro* cultured RPE cells were treated with or without autophagy inhibitor CQ, and a rescue experiment was performed by transfecting SOCS2-expressing plasmids. SOCS2^–/–^ RPE had less LC3B puncta and the rescued group showed increased LC3B puncta than SOCS2^–/–^ RPE (Figures 2C,D). The amount of p62 is negatively correlated with autophagic activity for its ultimate degradation by autophagy. The fluorescence intensity of p62 showed a slight difference (Figures 2C,E). The amount of LC3B and p62 was detected in cultured RPE cells by immunoblot (Figure 2F). Low LC3B-II/LC3B-I ratio was measured in SOCS2^–/–^ RPE, whereas the heightened ratio was measured in rescued group and CQ enlarged this ratio by blocking the degradation of LC3B-II (Figure 2G). More p62 was detected in SOCS2^–/–^ RPE compared to WT RPE and more p62 stagnated in SOCS2 rescued RPE than SOCS2^–/–^ RPE after CQ blocking autophagy (Figure 2H).

### Overexpression of Suppressor of Cytokine Signaling 2 Promotes Autophagy in ARPE-19

**FIGURE 3:**
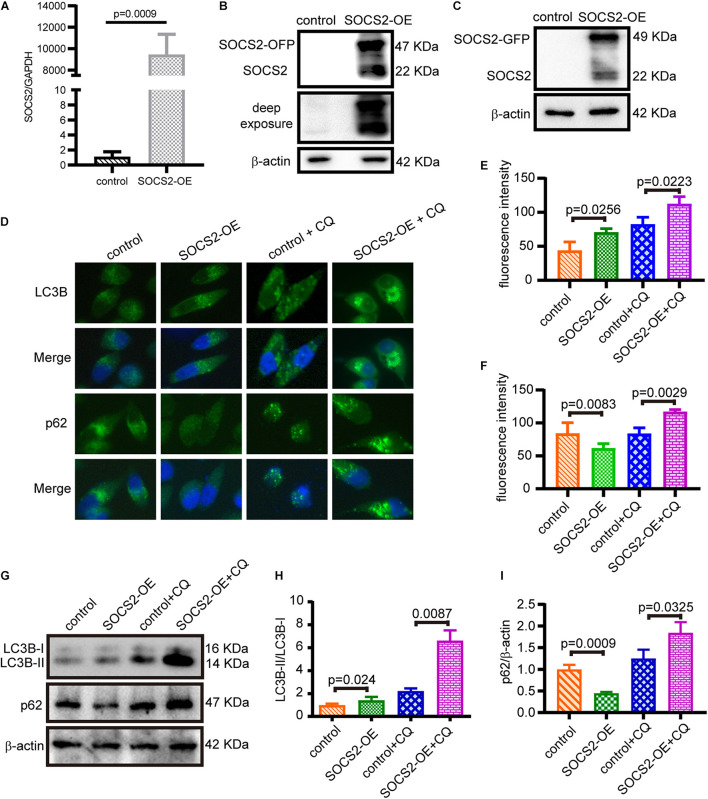
SOCS2 promoted autophagy in ARPE-19 cells. **(A)** The mRNA expression of SOCS2 was detected by real-time quantitative PCR in ARPE-19 after transient transfection with pCMV3-Human-SOCS2-OFP expression plasmids or control vectors. The two-tailed unpaired *t*-test was used. **(B,C)** Western blot images of SOCS2-OFP and SOCS2-GFP were detected by the SOCS2 antibody to check the overexpression of SOCS2. **(D)** LC3B puncta dots (green) and p62 puncta dots (green) were stained by immunofluorescence in ARPE-19 cells transfected with pCMV3-Human-SOCS2-OFP expression plasmids or control vectors for 24 h, then cells were starved and treated with or without 30 μM CQ for 24 h. **(E,F)** Fluorescence intensity of LC3B and p62 was quantified by ImageJ software. **(G)** Western blot detected the expression of LC3B and p62 in SOCS2 overexpressing ARPE-19 cells that were treated with or without 30 μM CQ for 24 h. **(H)** The ratio of LC3B-II/LC3B-I was quantified by ImageJ software and normalized to β-actin. Values for the control were set to 1. **(I)** p62 was quantified by ImageJ software and normalized to β-actin. Values for the control were set to 1. Statistical significance was determined by using the two-tailed unpaired *t*-test.

To confirm the function of SOCS2 in RPE, SOCS2 overexpression (SOCS2-OE) models were established. Two sets of expression plasmids were employed in ARPE-19 by transient transfection, pCMV3-SOCS2-OFP plasmids or control vectors, and pCMV3-SOCS2-GFP plasmids or control vectors. The mRNA expression of SOCS2 increased around 9,000-fold in the SOCS2-OE group (Figure 3A) and the protein expression of SOCS2-GFP and SOCS2-OFP was detected (Figures 3B,C). To confirm the role of SOCS2 in autophagy, autophagic activity was evaluated in the SOCS2-OE model by detecting the immunofluorescence of LC3B and p62. Notably, the SOCS2-OE group had more LC3B puncta and less p62 puncta compared to the control. More LC3B and p62 stagnated in the SOCS2-OE model compared to control after CQ blocking autophagy (Figures 3D–F). The ratio of LC3B-II/LC3B-I consistently increased significantly in the SOCS2-OE model after CQ treatment, whereas p62 decreased in the SOCS2-OE group and more p62 was detected in the SOCS2-OE group after CQ treatment (Figures 3G–I).

### Suppressor of Cytokine Signaling 2 Participates in a Ubiquitin-Autophagy-Lysosomal Pathway

**FIGURE 4:**
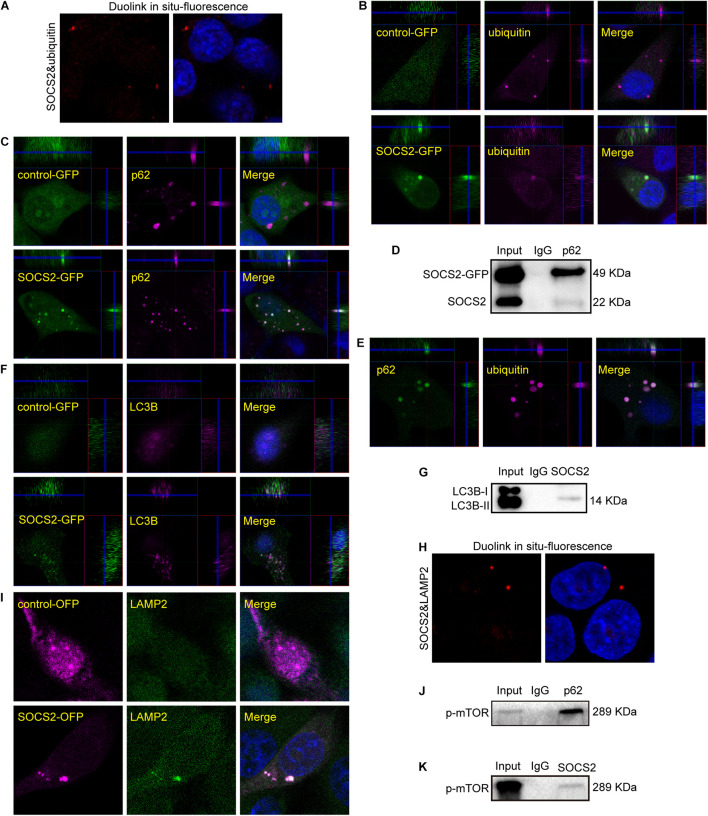
Ubiquitylated proteins recognized by SOCS2 were transferred into autophagosomes and autolysosomes. **(A)** The endogenous detection of protein interaction between SOCS2 and ubiquitin was detected in starved ARPE-19 by Duolink *in situ* fluorescence. The red fluorescence indicated the interaction of SOCS2 and ubiquitin. **(B)** Immunofluorescence staining with ubiquitin (red) and DAPI (blue) was performed in starved ARPE-19 with transient transfection of GFP control vectors or SOCS2-GFP plasmids. The yellow dots indicated the colocalization of SOCS2 and ubiquitin in merged pictures. **(C)** Immunofluorescence staining with the antibody p62 (red) and DAPI (blue) in ARPE-19 with the same treatment of B. The yellow dots indicated the colocalization of SOCS2 and p62 in merged pictures. **(D)** The immunoprecipitation of p62 and SOCS2 was detected in ARPE-19 with the same treatment of B. **(E)** Immunofluorescence staining was performed with the antibodies against p62 (green) and ubiquitin (red) in starved ARPE-19. **(F)** Immunofluorescence staining with the antibody LC3B (red) in ARPE-19 with the same treatment of B. **(G)** The immunoprecipitation of LC3B and SOCS2 was detected in ARPE-19 with the same treatment of B. **(H)** The endogenous detection of protein interaction between SOCS2 and LAMP2 was detected in starved ARPE-19 by Duolink *in situ* fluorescence. The red fluorescence indicated the interaction of SOCS2 and LAMP2. **(I)** Immunofluorescence staining with the antibody LAMP2 in ARPE-19 after transient transfection of SOCS2-OFP plasmids or OFP control vectors. **(J,K)** The immunoprecipitation of p62 and phosphorylated mammalian target of rapamycin (p-mTOR), SOCS2, and p-mTOR were detected in ARPE-19 with the same treatment of B.

As SOCS2 is a substrate recognition subunit of a ubiquitin ligase complex, to date, several proteins are identified as the substrates of SOCS2 such as GHR, erythropoietin receptor (EpoR), nuclear Dbf2-related kinase 1 (NDR1), and others (Vesterlund et al., 2011; Paul et al., 2017; Kung et al., 2019). Experiments of Duolink *in situ* fluorescence were performed in serum-starved ARPE-19 to detect the endogenous protein interaction of SOCS2 and ubiquitylated proteins. Red fluorescence indicated the colocalization of SOCS2 and ubiquitylated proteins (Figure 4A). To confirm the colocalization of SOCS2 and ubiquitylated proteins, either control-GFP or SOCS2-GFP was overexpressed in ARPE-19. The colocalization of SOCS2-GFP and ubiquitylated proteins were detected by the confocal microscopy with Z-stacked images (Figure 4B). The interaction of SOCS2-GFP and p62 was also detected by immunofluorescence and co-IP (Figures 4C,D). The p62 bodies were ubiquitin-containing protein aggregates (ubiquitin-positive inclusions) in the cytoplasm of ARPE-19 (Figure 4E). SOCS2 recognized the substrates for ubiquitination and p62 recruited ubiquitylated proteins for degradation. To check whether the ubiquitylated proteins recognized by SOCS2 were recruited to autophagosomes, the colocalization of SOCS2 and LC3B was detected by immunofluorescence and co-IP (Figures 4F,G).

After 24 h of CQ treatment, the colocalization of ubiquitin and a lysosome marker LAMP1 were reported (Zhan et al., 2016). To confirm the degradation of proteins identified by SOCS2 in the lysosome, experiments of Duolink *in situ* fluorescence were deployed to detect the endogenous protein interaction between SOCS2 and LAMP2 in ARPE-19. Red fluorescence indicated the colocalization of SOCS2 and LAMP2 (Figure 4H). To further identify the interaction of SOCS2 and LAMP2, SOCS2-OFP or control-OFP was expressed in ARPE-19 and the colocalization of SOCS2-OFP and LAMP2 was detected by confocal microscopy (Figure 4I). Earlier studies showed that the p62-mTOR complex translocated to the lysosome membrane in response to amino acid deprivation (Duran et al., 2011). In serum-starved ARPE-19, the interaction of p62 and p-mTOR was detected (Figure 4J). As additional evidence of SOCS2 locating in the lysosome, the interaction of SOCS2 and p-mTOR was also detected in starved ARPE-19 (Figure 4K). These findings imply that SOCS2 may directly participate in the ubiquitin-autophagy-lysosomal pathway.

### Suppressor of Cytokine Signaling 2 Enhances Autophagy by Regulating the Phosphorylation of Glycogen Synthase Kinase 3β and Mammalian Target of Rapamycin in Retinal Pigment Epithelium

**FIGURE 5:**
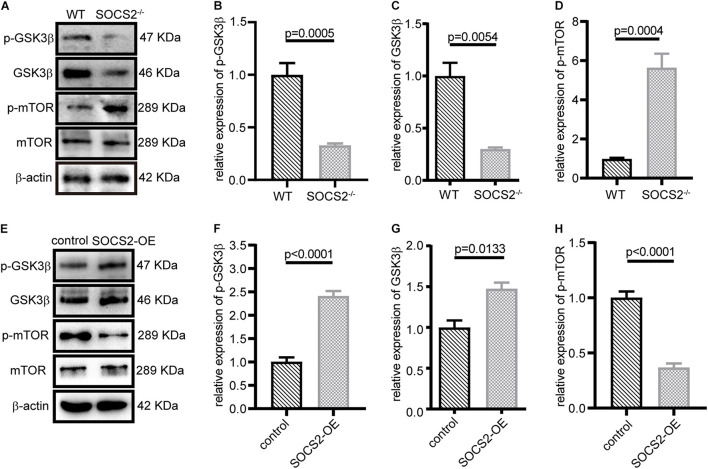
SOCS2 regulated the phosphorylation of glycogen synthase kinase 3β (GSK3β) and mTOR. **(A)** Western blot images of p-GSK3β, GSK3β, and p-mTOR were detected in the primary RPE from WT and SOCS2^–/–^. **(B,C,D)** Data shown are the mean of GSK3β, p-GSK3β, and p-mTOR bands quantified by ImageJ software and normalized to β-actin. Values for the control were set to 1. **(E)** Phosphorylation of GSK3β and mTOR was detected by immunoblot in ARPE-19 after transient transfection with SOCS2-OFP expression plasmids or OFP control vectors. **(F,G,H)** Data shown are the mean of p-GSK3β, GSK3β, and p-mTOR bands quantified by ImageJ software and normalized to β-actin. The two-tailed unpaired *t*-test was used.

Suppressor of cytokine signaling 2 negatively regulates growth hormone signaling. GSK3β is inhibited in response to insulin or growth factors and negatively mediated activation of mTOR, but the relationship between SOCS2 and GSK3β has not been yet revealed. The expression of p-GSK3β (Tyr216) and GSK3β decreased (Figures 5A–C), whereas the phosphorylation of mTOR (Ser2448) significantly increased in SOCS2^–/–^ RPE (Figure 5D). By contrast, SOCS2 overexpression increased the expression of GSK3β and p-GSK3β (Figures 5E–G), whereas the expression of p-mTOR decreased in the SOCS2-OE model (Figure 5H). These results imply that SOCS2 may enhance autophagy by regulating the phosphorylation of GSK3β and mTOR, both of which are involved in the regulation of autophagy.

## Discussion

The decreased autophagic ability of RPE with age is an important reason for drusen formation. Many studies reported that promoting autophagy protects RPE and contributes to maintaining retinal metabolism. The CD36 ligand-promoted autophagy protects RPE from oxidative stress (Dorion et al., 2021). The transcription factor EB (TFEB) inducer trehalose protects RPE against oxidative damage and cell death by upregulating autophagy (Abokyi et al., 2020). Metformin defenses hydrogen peroxide-induced oxidative damage in RPE by enhancing autophagy through activation of the AMP-activated protein kinase (AMPK) pathway (Zhao et al., 2020). Two recent studies described that SOCS2 is involved in autophagy in myocardial I/R and Huntington’s disease (Li et al., 2019; Cho et al., 2021), but the underlying molecular mechanisms for SOCS2 regulating autophagy are unclear. In this study, we explored that SOCS2 is the enhancement of autophagy in RPE by regulating mTOR and GSK3β.

Signal duration for the activated GHR is mainly controlled by SOCS2 (Sarajlic et al., 2019). The deletion of SOCS2 leads to overgrowth and abnormal metabolism, both of which may contribute to the accumulation of basal deposits between the RPE and Bruch’s membrane. As SOCS2^–/–^ mice are much heavier compared to the WT littermates, weight gain may be due not only to GH/insulin-like growth factor signaling but also to the accumulation of metabolic waste that cannot be removed properly by impaired autophagy. Basal deposits lead to structural and functional abnormity of RPE such as the blood-retinal barrier, phagocytosis, and light absorption, while RPE dysfunction accelerates the formation of basal deposits. This vicious cycle drives the damage to RPE.

The suppressor of cytokine signaling 2 is a substrate recognition subunit of a Cul5 E3 ubiquitin ligase complex. In this study, we found that SOCS2 colocalized not only with ubiquitylated proteins, but also with p62, LC3, and LAMP2 during autophagy. Ubiquitylated proteins were recruited by p62 to autophagosomes. LC3 is the most well-characterized autophagosome marker in mammalian cells. p62 binding directly to LC3 *via* a short LC3-interacting region (LIR) that serves as a mechanism to deliver autophagic cargo for degradation (Xu et al., 2014). LC3 binds to the crucial Trp/Phe/Tyr-X-X-Leu/Ile/Val (W/F/YXXL/I/V) motif in the LIR of proteins (Qiu et al., 2017; Sun et al., 2017). Four putative LIRs (W/F/YXXL/I/V) motifs were identified in the SOCS2 peptide sequence, implying that SOCS2 may directly bind with LC3 in the formation of autophagosomes.

Glycogen synthase kinase 3β is a serine/threonine kinase that mediates the inactivation of glycogen synthase and is inhibited by insulin signaling. Many studies reported that GSK3β is involved in autophagy. For example, GSK3β induces autophagy by phosphorylating ULK1 (Ryu et al., 2021) and C42 promotes autophagy by regulating the K-Ras/GSK3 signaling pathway in HCT116 cells (Niu et al., 2014). Inhibition of GSK3β stimulated mTOR signaling and inhibited autophagy through a rapamycin-sensitive mechanism during myocardial ischemia and IR (Zhai et al., 2011). Nimbolide negatively regulates PI3K/Akt signaling with a consequent increase in p-GSK3β that inhibits autophagy (Sophia et al., 2018). GSK3β activation of mTOR inhibits autophagic and lysosomal activity in neurons (Rippin and Eldar-Finkelman, 2021). Ischemic post-conditioning ameliorates intestinal IR injury by evoking autophagy and inactivating GSK3β/nuclear factor erythroid 2-related factor 2 (Nrf2) pathway (Chen et al., 2020). However, none of the aforementioned studies identified the relationship between SOCS2 and GSK3β. We found that SOCS2 enhanced GSK3β activity in RPE. Low GSK3β expression was observed in SOCS2^–/–^ RPE, which showed impaired autophagy. Conversely, overexpression of SOCS2 increased the activity of GSK3β and enhanced autophagy. A high positive correlation was observed between SOCS2 and GSK3β in RPE. We also found that SOCS2 negatively regulated mTOR, which is one of the pivotal factors for the regulation of autophagy. The overexpression of SOCS2 significantly inhibited the phosphorylation of mTOR. By contrast, RPE lacking the *SOCS2* gene showed much higher levels of phosphorylation of mTOR. Therefore, we concluded that SOCS2 is the enhancement of autophagy that may occur through the regulation of GSK3β and mTOR.

Loss of SOCS2 results in autophagic clearance defects due to inactive GSK3β and active mTOR signaling. Defective autophagy of RPE leads to detrimental aggregation, which is one of the clinical hallmarks of AMD. SOCS2^–/–^ mice exhibit abnormal RPE-Bruch’s membrane-choriocapillaris complex (Figure 1). It implies that defective autophagy is an important mechanism in the pathogenesis of AMD. Thus, identifying molecular targets that can regulate autophagy provides a new avenue for the treatment of AMD. Since the expression of SOCS2 were detected in the ONL of retina, and the SOCS2^–/–^ mice show abnormal arrangement of the photoreceptor ONL, we speculate that SOCS2 is also involved in photoreceptor dysfunction and AMD pathology, but further experiments are needed in the future study. To date, AMD is an incurable disease, especially for dry AMD. Explored molecular mechanisms and possible therapeutic targets are a top priority. In this study, we performed both *in vitro* and *in vivo* experiments to investigate the role of SOCS2 in autophagy and found that SOCS2 participates directly in autophagy and enhances autophagy by regulating the phosphorylation of GSK3β and mTOR. This study outlined the process of SOCS2 involving autophagy and provided deeper insights into the molecular mechanisms, which may develop a potential therapeutic target for AMD.

## Data Availability Statement

The original contributions presented in the study are included in the article/supplementary material, further inquiries can be directed to the corresponding author.

## Ethics Statement

The animal study was reviewed and approved by the Institutional Animal Care and Use Committee of Wenzhou Medical University.

## Author Contributions

Z-LC and X-YL investigated and designed the research and drafted the manuscript. Z-LC, X-YL, and GC analyzed the data. Z-LC, R-HW, and X-YL contributed to funding acquisition. X-YL, RL, JC, JW, R-HW, and H-MQ performed research. All authors contributed to the article and approved the submitted version.

## Conflict of Interest

The authors declare that the research was conducted in the absence of any commercial or financial relationships that could be construed as a potential conflict of interest.

## Publisher’s Note

All claims expressed in this article are solely those of the authors and do not necessarily represent those of their affiliated organizations, or those of the publisher, the editors and the reviewers. Any product that may be evaluated in this article, or claim that may be made by its manufacturer, is not guaranteed or endorsed by the publisher.
